# Foreign Object Detection by Sub-Terahertz Quasi-Bessel Beam Imaging

**DOI:** 10.3390/s130100071

**Published:** 2012-12-20

**Authors:** Gyeongsik Ok, Sung-Wook Choi, Kyung Hyun Park, Hyang Sook Chun

**Affiliations:** 1 Food Safety Research Group, Korea Food Research Institute, Backhyun 516, Sungnam, Gyeonggi-do 463-746, Korea; E-Mails: ocknet88@naver.com (G.O.); swchoi@kfri.re.kr (S.-W.C.); 2 THz Photonics Creative Research Center, ETRI, Daejeon 305-700, Korea; E-Mail: khp@etri.re.kr

**Keywords:** terahertz imaging, sub-terahertz imaging, quasi-Bessel beam, axicon, foreign object detection, FDTD

## Abstract

Food quality monitoring, particularly foreign object detection, has recently become a critical issue for the food industry. In contrast to X-ray imaging, terahertz imaging can provide a safe and ionizing-radiation-free nondestructive inspection method for foreign object sensing. In this work, a quasi-Bessel beam (QBB) known to be nondiffracting was generated by a conical dielectric lens to detect foreign objects in food samples. Using numerical evaluation via the finite-difference time-domain (FDTD) method, the beam profiles of a QBB were evaluated and compared with the results obtained via analytical calculation and experimental characterization (knife edge method, point scanning method). The FDTD method enables a more precise estimation of the beam profile. Foreign objects in food samples, namely crickets, were then detected with the QBB, which had a deep focus and a high spatial resolution at 210 GHz. Transmitted images using a Gaussian beam obtained with a conventional lens were compared in the sub-terahertz frequency experimentally with those using a QBB generated using an axicon.

## 1. Introduction

The detection of foreign objects during the food inspection process is critical to ensuring the quality of food products. Many techniques have been developed commercially and used for the detection and identification of foreign objects during the manufacturing and packaging of food products. Metal detectors, optical detectors, and X-ray systems are in widespread use in the food industry, because they are cost-effective and reliable. Moreover, X-ray systems can reveal items made of hard nonmetallic materials such as stone, glass, bone, rubber, and plastic when embedded in food products. However, for soft materials (often organic materials) inside food products, accurate identification by X-ray systems is known to be difficult [1].

Unlike X-ray radiation with its ionizing properties, sub-terahertz and terahertz radiation (from 0.1 THz to 10 THz) of low photon energy is nonionizing, making it suitable for investigating biological materials, human-body-related security, and food products [2–4]. Despite the fact some nonmetallic and dry materials are opaque to visible and infrared light, they are transparent in the terahertz frequency range because of their low absorption. Terahertz technology can therefore offer a novel quality-inspection method, particularly for food, that offers advantages to conventional noninvasive and nondestructive methods. More specifically, terahertz spectroscopic imaging (terahertz time-domain-spectroscopic imaging) and monochromatic imaging (continuous-wave (CW) imaging) have been recognized as promising methods for nondestructive quality inspection in terahertz applications [5–7].

Since terahertz imaging was first demonstrated by Hu and Nuss [5], there have been many technical improvements and developments in various aspects of imaging performance, following the increased variety of sources and detectors for terahertz systems [8]. However, there remain limitations such as spatial resolution, depth of focus, acquisition speed, and detection area. To address these technical hurdles in terahertz imaging, various approaches have been suggested and demonstrated [9]. In particular, achieving subwavelength resolution at terahertz frequencies has been sought by using near-field techniques [10]. However, because fine lateral spatial resolution and deep depth of focus cannot simultaneously be obtained by conventional imaging methods, a Bessel beam known as a “nondiffracting” beam should be introduced to enable the practical use of terahertz imaging in industry.

A nondiffracting beam described by the zeroth-order Bessel function of the first kind, *J_0_*, was proposed by Durnin *et al.* in 1987 [11]. It has the characteristic that its lateral electric field distribution is invariant along the axis. An ideal Bessel beam of infinite transverse extent cannot be created in practice, but a quasi-Bessel beam (QBB) over a finite range can be realized. An approximate nondiffracting beam can be generated by various methods, such as by using an extremely narrow annular aperture on the lens pupil, computer-generated holograms, or a conical lens known as an axicon [12–14]. It has been reported that an axicon can offer high-intensity QBB [15]. Although such a nondiffracting beam would be attractive for noninvasive imaging applications, this has been rarely reported in terahertz imaging or in food-inspection applications [16,17].

Using the non-diffracting nature of Bessel beams, we demonstrate, for the first time to our knowledge, the sub-terahertz imaging of organic foreign objects embedded in food samples. To characterize the QBB, we performed theoretical and numerical calculations and compared them with results obtained by the knife-edge method and the point scanning method. The image of a Gaussian beam focused by a conventional aspherical lens is compared experimentally with that for a sub-terahertz QBB using an axicon. To acquire the two-dimensional (2D) image of the sample, a CW terahertz imaging method with point-to-point scanning was used as an imaging tool. These results show that terahertz imaging by QBB can offer better opportunities for nondestructive food inspection.

## 2. Theory and Numerical Calculations

Lateral spatial resolution in terahertz imaging by point-to-point scanning is determined primarily by the spot diameter at a focal point, which depends on the waist and wavelength of the incident Gaussian beam [18]. Moreover, the waist of the Gaussian beam in the lens system is affected by the geometry of the lens system and by lens parameters such as focal length, diameter, and spacing. If these parameters can be optimized, thereby acquiring a minimum focal spot size, diffraction-limited resolution can be obtained. However, the Rayleigh range (half of the depth of focus) calculated by *π*·*w^2^_0_*/*λ* is also minimized in this approach, where *w_0_* is the waist and *λ* is the wavelength of the incident Gaussian beam. To overcome this limitation, a QBB using an axicon for terahertz imaging should be introduced as described above.

Therefore, the beam profile given by the field intensity distribution should first be calculated for terahertz imaging. The results of this calculation can then provide useful information, such as the depth of focus and spot diameter. In general, the field intensity distribution of the QBB in the image space can be calculated using scalar diffraction theory or vector diffraction theory. In this work, the results of analytical calculations using scalar diffraction theory are compared with those of the finite-difference time-domain (FDTD) method. Using these two methods, the beam profile of the QBB beam is evaluated numerically as follows.

### 2.1. Spatial-Intensity Distribution Function for a QBB

The beam profile of a QBB can be calculated by the field-intensity distribution function behind an axicon, which is illuminated with a Gaussian beam. For obtaining the analytical expression of the intensity distribution behind an axicon, various approaches have been reported ([15], and references therein). In this work, based on scalar diffraction theory, the intensity distribution function given by the work of Oto Brzobohatý *et al.* was used [15]. Even though it was derived by using appropriate approximations for the functions in visible frequencies, its applicability to the terahertz applications can be verified with following the intensity distribution function in cylindrical coordinates, which is given by [19]:

(1) $$I\left(\rho ,z\right)=\frac{4\text{Pk}\sin\alpha_{0}}{w_{0}}\frac{z}{z_{\max}}\exp\left(-\frac{2z^{2}}{z_{\max}^{2}}\right)J_{0}^{2}\left(k\rho \sin\alpha_{0}\right)$$

**Figure 1.**
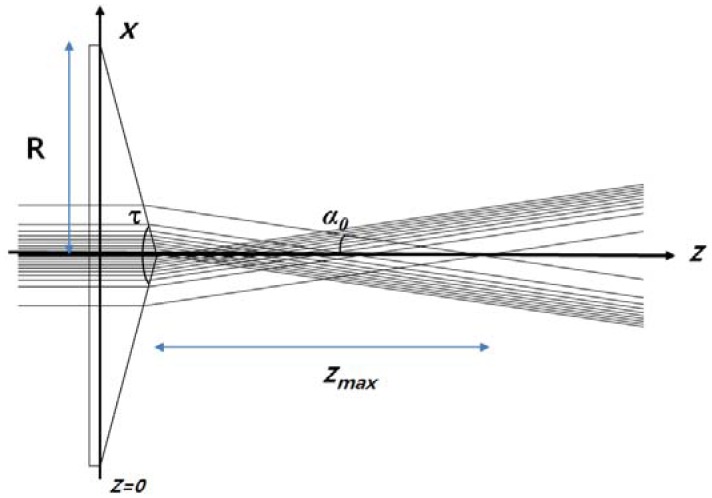
Ray model of a QBB and an axicon illuminated by a Gaussian beam (2*R* denotes the axicon diameter of 50 mm, *τ* is the 150° apex angle of the axicon, *α_0_* is the 8.5° semiapex angle).

Here, *P* is the total power of the incident Gaussian beam and *k* = *2π*/*λ* is the angular wavenumber, *w_0_* is the waist of the incident Gaussian beam, *α_0_* is the semiapex angle (see Figure 1), *z_max_* (depth of focus) is the nondiffracting region (=*w_0_*/tan*α_0_*) where the Bessel beam exists, and *ρ* denotes the radial distance from the optical axis *z*. Using the fact that the first zero of a zeroth-order Bessel function is at *kρ*sin*α_0_* = 2.4048, the radius of the central beam spot is given by 2.4048/(*k*sin*α_0_*). In particular, *α_0_* is related to the axicon parameters by:

(2) $$\alpha_{0}=\arcsin\left(\frac{n}{n_{0}}\cos\left(\frac{\tau }{2}\right)\right)+\frac{\tau -\pi }{2}$$

Here, *n* and *n_0_* are the refractive indices of the axicon (*n* = 1.54) and the surrounding medium (*n_0_* = 1.0), respectively, and *τ* is the apex angle of the axicon (150°) [15]. With these axicon parameters and the relations described above, the nondiffracting region *z_max_* is calculated as 40.2 mm and the radius of central beam spot is 3.7 mm (*w_0_* = 6 mm), as described in Figure 1. Note that, in this calculation, the waist of the incident Gaussian beam is located at *z* = 0 (flat wavefront at *z* = 0), and the apex of the axicon is located at *z* = *R*/tan(2/*τ*) = 6.7 mm. And we also can calculate theoretically the beam profile (spatial 2D intensity distribution) of the QBB at a sub-terahertz frequency (210 GHz) using the above equations in following section.

### 2.2. Comparison with FDTD Calculation

**Figure 2.**
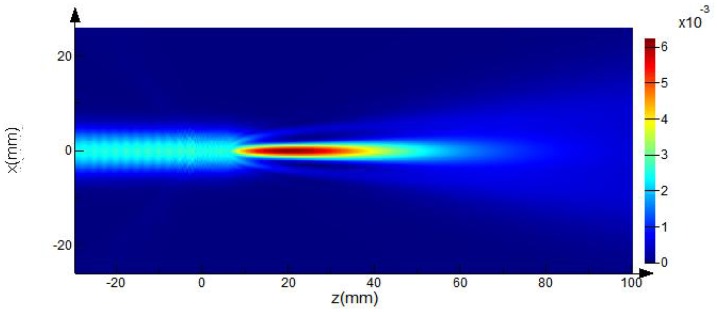
2D intensity distribution of a QBB calculated by the FDTD method using the parameter values given in the text.

**Figure 3.**
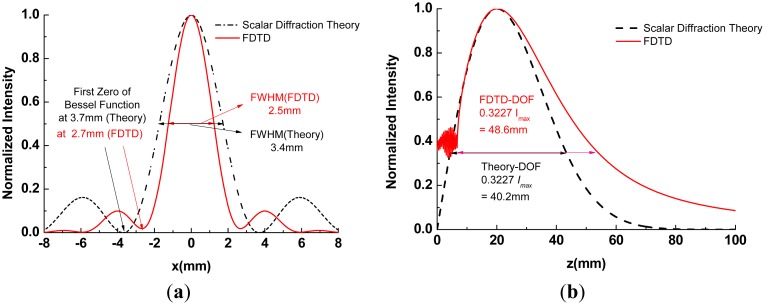
(**a**) Radial intensity distribution for the central spot of the QBB at z = *z_max_*/2 (the maximum of the on-axis intensity occurs at this point); (**b**) Axial intensity distribution behind the axicon for the QBB (on-axis intensity profile at *ρ* = 0).

Because of the rotational symmetry of the axicon, the 2D FDTD simulation was performed with a commercial package (Lumerical FDTD solutions, Vancouver, BC, Canada) [20]. Perfectly matched layers were used at all boundaries and a Gaussian beam was used as the excitation source at a frequency of 0.21 THz. The beam waist of 6.0 mm was located at *z* = 0. The source was linearly polarized in the XZ plane. Figure 2 shows the 2D intensity distribution for a QBB generated by an axicon with the parameter values given above. In particular, the results of the analytical calculations using Equations (1) and (2) are compared with those for the FDTD method and are shown in Figure 3, as a cross-section plot of Figure 2.

Although the scale of the axicon diameter is one order of magnitude greater than the wavelength of beam, performance degradation does not appear at 210 GHz, as shown in Figures 2 and 3. Furthermore, the diffraction effect caused by the outer edges and the influence of the slightly rounded tip can be neglected because the waist of the incident beam is less than one fourth of the axicon diameter and the imperfection at the axicon tip is smaller than the beam wavelength at the terahertz frequency [15].

According to the theoretical calculations above, the radius of the beam defined by the first zero point of the Bessel function is 3.7 mm, and the depth of focus defined by 0.3227 *I_max_* (maximum on-axis intensity) is 40.2 mm, as shown in Figure 3 [19]. However, the results of the FDTD calculations show that the radius of the beam is 2.7 mm and the depth of focus is 48.6 mm. Furthermore, the on-axis intensity cannot physically be equal to zero at *z* = 0, as shown in Figure 3(b). As Cižmár pointed out in his thesis, these inaccuracies can be caused by an asymptotic approximation of the Bessel function $\left(J_{0}\;\left(x\right)\approx \sqrt{2\;/\;\pi x}\cos\left(x-\pi \;/\;4\right)\right)$ used in the calculation of the spatial-frequency spectrum, for obtaining the previous function (Equation (1)) [19]. These results reveal that the previous analytical model for QBB using an axicon illuminated by a Gaussian beam should be modified in terahertz frequencies.

## 3. Experimental Section

### 3.1. Material Preparations

**Figure 4.**
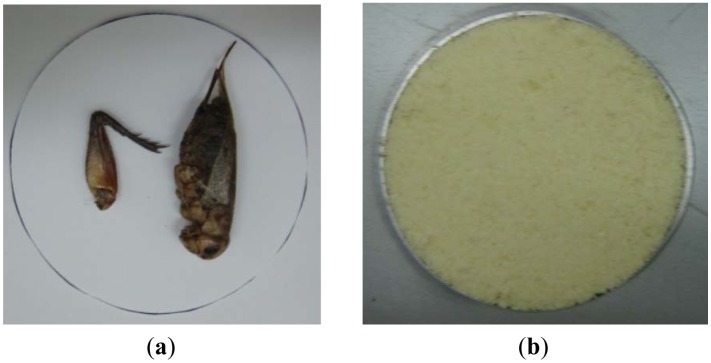
(**a**) Photographic image of the crickets; (**b**) Photographic image of the crickets embedded in noodle flour.

As described in a previous study, two foreign objects, namely crickets, were used. They were 35 mm and 50 mm in length, and 5.5 mm and 7 mm in thickness, respectively (Figure 4) [1]. Instant noodles (*ramyun* or *ramen*, local name of instant noodles) were the material selected to be the test food product, given its widespread consumption in South Korea, and one where several instances of foreign-body contamination have been found. The noodles were ground into fine flour in a blender until the sample could pass through a No. 20 sieve. The noodle flour was then used to fill a 50-mm-diameter circular hole, formed in an aluminum composite panel (100 mm × 100 mm × 8.5 mm). After embedding the crickets into the flour, both sides of the sample holder were covered with a thin acrylic sheet (thickness 100 μm).

### 3.2. Experimental Setup

**Figure 5.**
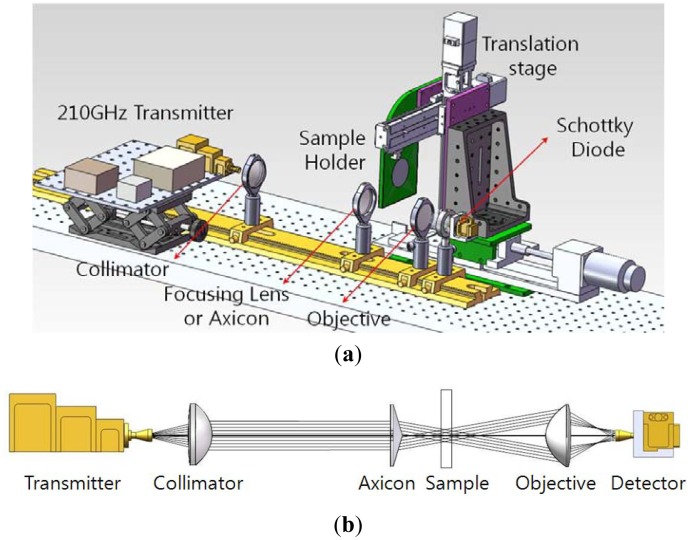
(**a**) CW terahertz imaging setup using an axicon (3D rendering image). (**b**) Optics layout for CW terahertz imaging. For Gaussian beam illumination, an axicon can be changed with the TPX lens.

The transmission geometry for acquiring the terahertz images is shown in Figure 5. A 210-GHz transmitter (Virginia Diodes Inc., Charlottesville, VA, USA) with an output power of 75 mW was used as the CW terahertz source. The E-field from source was plane-polarized and perpendicular to the underlying optical table. The detector was a Schottky diode (Virginia Diodes Inc.) with a responsivity of 2000 V/W (typically) and a WR-5 conical horn antenna was attached. The sample holder described above was mounted on a motorized 3D translation stage to enable acquisition of a scanned image. Point-to-point scanning by the motorized stage (in two axes) was performed with a step resolution of 0.5 mm, over a range of 80 mm × 60 mm (160 × 120 pixels, measure time: 45 min/frame).

A collimated Gaussian beam from the first lens is directed to a second lens for Gaussian beam focusing or to an axicon for QBB focusing. Two aspherical TPX lenses (n = 1.465; Zomega Terahertz Corp., East Greenbush, NY, USA) were used, with the same focal length and diameter (f = 38 mm, D = 50 mm). Here, the focal lengths of the TPX lenses were recalculated for 210-GHz frequencies using ray-tracing software and the lens data obtained from Zomega Terahertz Corp. (East Greenbush, NY, USA)The axicon was designed with the parameter values described above and fabricated in high-density polyethylene (HDPE, n = 1.54). The focused beams were transmitted through the sample and collected by a detector with an aspherical objective (HDPE, f = 23 mm, D = 50 mm). Particularly, it should be noted that the objective lens can focus the diverging rays onto the detector in Figure 5(b).

The analog signal from the detector was amplified (gain = 1,000) and filtered (low-pass filter) by a low-noise preamplifier (SR 560; Stanford Research Systems, Sunnyvale, CA, USA). Finally, the digitized data acquired by an NI DAQ board (National Instruments, Austin, TX, USA) were fed into a personal computer and averaged over time (number of averages = 2,000).

## 4. Results and Discussion

### 4.1. Beam Characterization by the Knife-Edge Method

**Figure 6.**
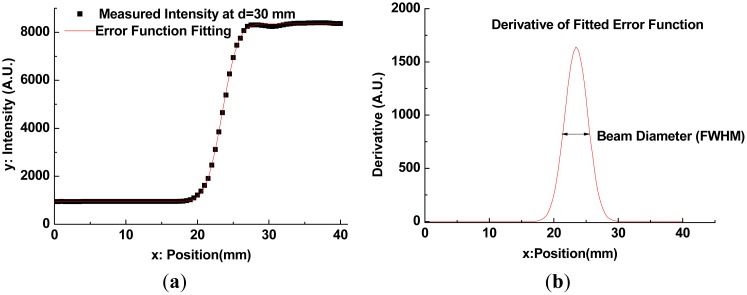
(**a**) Measured intensity profile of QBB using the knife-edge method (d = 30 mm) and curve fitting; (**b**) Derivative of fitted error function and determination of the beam diameter (FWHM).

**Figure 7.**
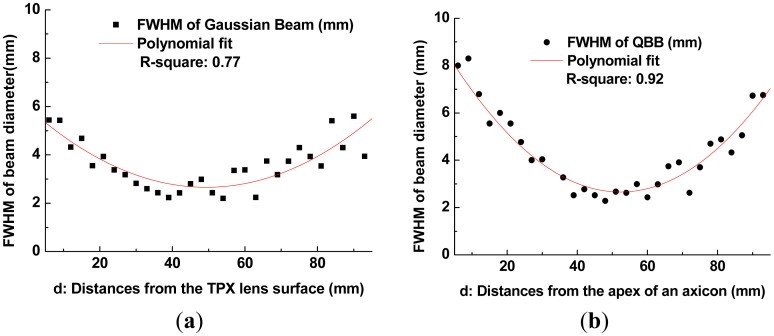
(**a**) Gaussian beam-width profile using the knife-edge method; (**b**) QBB width profile using the knife-edge method.

By using the measurement setup shown in Figure 5 and a razor blade with a sharp edge, the intensity profiles and beam profiles shown in Figures 6 and 7 were obtained. The razor blade was mounted on a wide metal sheet (thickness = 1 mm) on the motorized stage, and moved in a direction perpendicular to the propagation direction of the terahertz beams [21]. Each intensity profile was recorded while varying the z-axis position over a range of 0 mm (near the flat lens surface or the apex of an axicon) to 100 mm, in steps of 3 mm. Measured intensity profiles of both Gaussian beam and QBB can be fitted with the error function of sigmoid shape (Equation (3), *P_T_*(*x*)) and thereafter the derivative of fit function results in the Gaussian function [22]. It can give us the full width at half-maximum (FWHM) of beam diameter at certain z-position behind the lens. Here, *P_T_*(*x*) as a function of *x* (perpendicular to the *z* direction) is given by:

(3) $$P_{T}\;(x)=\frac{P_{0}}{2}[1+\text{erf}(\frac{\sqrt{2}\left(x-x_{0}\right)}{r})]+c$$

where *P_0_* is the total power intensity, *x_0_* is the center of plot, *r* is the 1/*e*^2^ radius of the beam, *c* is the baseline constant, and *erf* corresponds to the error function. For calculation of curve fitting and its derivative, we use OriginPro 8.0 software (OriginLab. Corp., Northampton, MA, USA). The curve fitting example for intensity profile of QBB at d = 30 mm is illustrated in Figure 6, where d denotes the distance from the TPX lens surface or the apex of an axicon to the razor blade. From these results, the FWHM of beam diameter at each z-position (corresponds to d) behind the lens can be obtained, as shown in Figure 7.

The red lines in Figure 7 show the second-order polynomial fits to each set of FWHM data. Using these fitting curves, the minimum beam width is estimated as 2.65 mm for the Gaussian beam and 2.66 mm for the QBB. Particularly for the QBB, it should be noted that the measured beam-width profile could be displayed differently with the results based on the 2D intensity distribution described in Figure 2. This discrepancy can be explained as follows. As illustrated in previous optics layout (Figure 5(b)), all rays from the axicon can arrive at a detector in this configuration, except the cases of scattering and diffraction. If the razor blade obstructs the optical beam path partially, detected beam intensity may be proportional to the unblocked fraction of beam cross-section. Thus, transmitted beam intensity can be only monitored with a detector, whether the interference of incident beam is occurred within central core or not. It means that the beam profile of axicon by knife edge method can be simply explained by ray tracing model as shown in Figure 1 and Figure 5(b). However, it is difficult to obtain the quasi Bessel beam distribution like the results of FDTD calculation in this configuration, because the position of detector is located out of the interference range. This is the reason why the beam profile of QBB by knife edge method is different with the previous results of FDTD calculation. For obtaining more similar beam profile of QBB like Figure 2, the point scanning method by varying the position of detector should be used [23]. Using the point scanning of a detector, local field distribution (ring pattern) generated by the interference of incident beam can be measured as following section.

### 4.2. Beam Characterization by the Point Scanning Method

**Figure 8.**
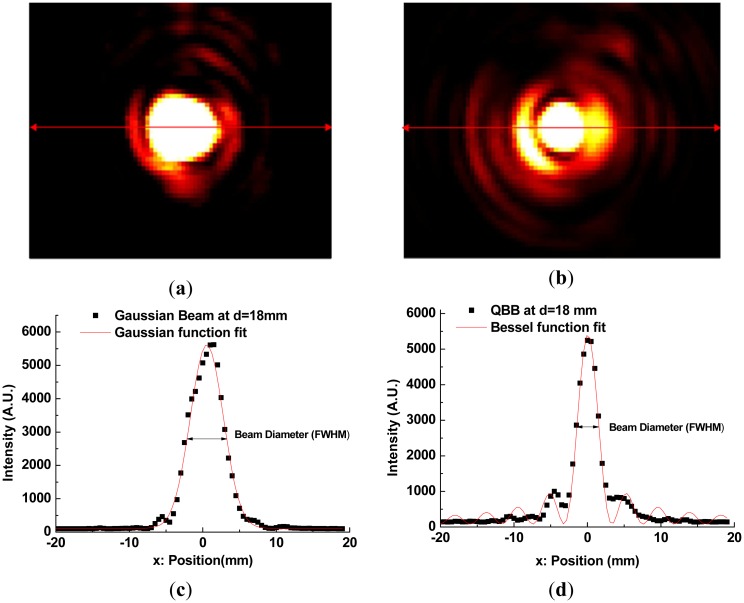
(**a**) 2D Image of Gaussian beam intensity profile by the point scanning method; (**b**) 2D Image of QBB intensity profile by the point scanning method; (**c**) Curve fitting of radial intensity distribution of Gaussian beam; (**d**) Curve fitting of radial intensity distribution of QBB. Both radial intensity profiles (**c**,**d**) were obtained from the image (**a**,**b**) measured at d = 18 mm.

**Figure 9.**
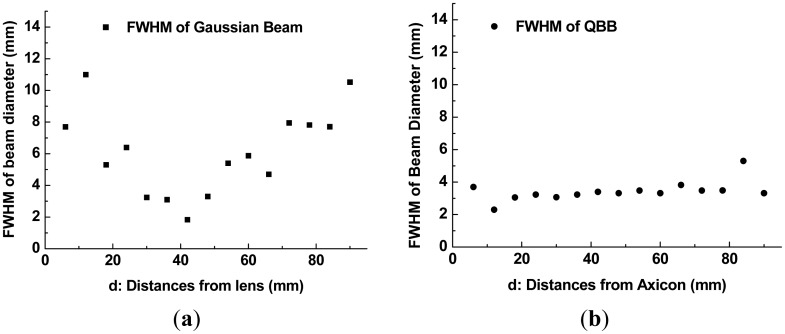
(**a**) Gaussian beam-width profile by the point scanning method; (**b**) QBB width profile by the point scanning method.

**Figure 10.**
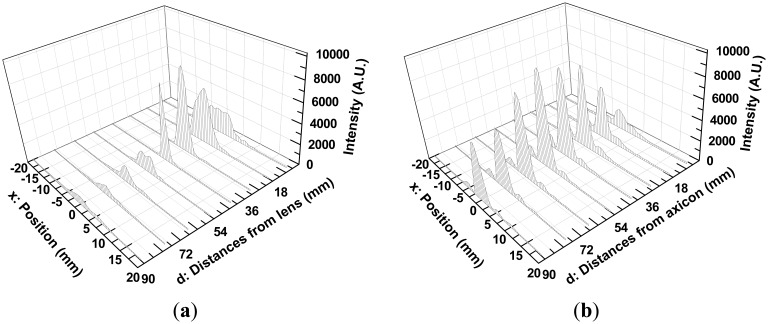
(**a**) Gaussian beam propagation after a TPX lens; (**b**) QBB propagation after an axicon. Both beam propagation plots are illustrated with the radial intensity distribution measured by the point scanning method.

For implementing point scanning with a single detector, a 3 mm pinhole in front of the detector was used, and the detector was mounted on a *X-Y-Z* translation stage, as describesd in Figure 5(a). At a specific z position, a 2D image of the intensity profile was obtained directly by point scanning (30 mm × 40 mm, 60 × 80 pixels) on the *X-Y* plane. Moreover, by varying the z-axis position over a range of 6 mm (near the flat lens surface or the apex of an axicon) to 90 mm, in steps of 6 mm, 15 intensity profile images were measured. Those images were used to determine the beam diameter (FWHM) as depicted in Figures 8 and 9, and finally to visualize the beam propagation as shown in Figure 10.

For example, Figure 8(a,b) show the images of intensity profile of Gaussian beam and QBB at d = 18 mm position, respectively. Here, d is the distance from the TPX lens surface or the apex of an axicon to the pinhole. The cross-section plots of the images at maximum intensity (red line in Figure 8(a,b)) are depicted in Figure 8(c,d). They can be numerically fitted with Gaussian function and Bessel function described previously, in order to determine the beam diameter (FWHM).

With these beam diameters and radial intensity distributions obtained from the images of intensity profile, finally, one can obtain the plots of beam diameter (beam-width) as a function of distance from a lens or an axicon to pinhole, as illustrated in Figure 9(a,b). Beam propagation over a long range can be visualized as illustrated in Figure 10(a,b).

As depicted in Figure 8(a,b), non-uniform intensity distributions of incident beam (not a perfect Gaussian beam) provide slightly distorted and noisy patterns to intensity profiles. They also affect the estimation of FWHM of beam diameter by curve fitting. Even though there are these unwanted disturbance in intensity profiles obtained by the point scanning method, Figure 9(a) shows a similar tendency with the beam width profile of Gaussian beam described in Figure 7(a).

However, the beam profile of QBB by the point scanning method (Figure 9(b)) shows a nearly flat profile over a long range, which is different with the result by the knife edge method (Figure 7(b)). This means that the QBB profile obtained by the point scanning method can demonstrate local intensity distribution more visibly than the knife edge method. Furthermore, these results are agreement with those of FDTD calculation, except longer depth of focus and wider spatial resolution than the results of FDTD calculation. Figure 10 visualizes these characteristics of QBB propagation obviously.

**Figure 11.**
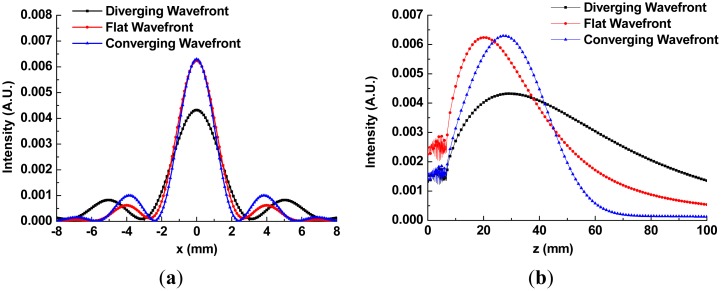
(**a**) The comparison of radial intensity distributions for the central spot of the QBBs at maximum of the on-axis intensity; (**b**) The comparison of axial intensity distributions behind the axicon for the QBBs. Here, the QBBs with different wavefronts at beam injection plane (*z* = 0 in Figure 1) were compared, using the FDTD method. The waist position of incident beam is *z* = −100 mm in diverging case, and *z* = 100 mm in converging case.

The inconsistency between the results of FDTD and those of point scanning can be explained by the following additional simulation results. The assumption used in both calculations of beam profiles was based on the fact that the wavefront in *z* = 0 plane was flat, as described previously. However, if the wavefront shape in *z* = 0 plane might be changed, the QBB propagation could be distorted as illustrated in Figure 11.

Particularly, if the waist position of incident beam is shifted to a negative direction of z, the wavefront in z = 0 plane has a diverging shape. The QBB with the diverging wavefront at z = 0 shows that the depth of focus becomes deeper, and the spatial resolution becomes wider in Figure 11. Thus, those disagreements of QBB profile between Figure 2 (calculation) and Figure 9 (experiment) can be explained by the wavefront shape in injection plane. It means that even if the incident Gaussian beam onto an axicon was collimated, the wavefront in *z* = 0 plane cannot be flat but diverging.

### 4.3. Improved Foreign Object Inspection by QBB

**Figure 12.**
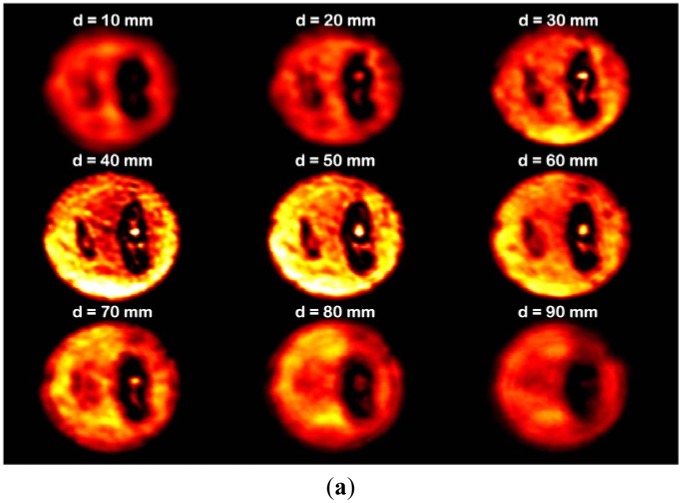

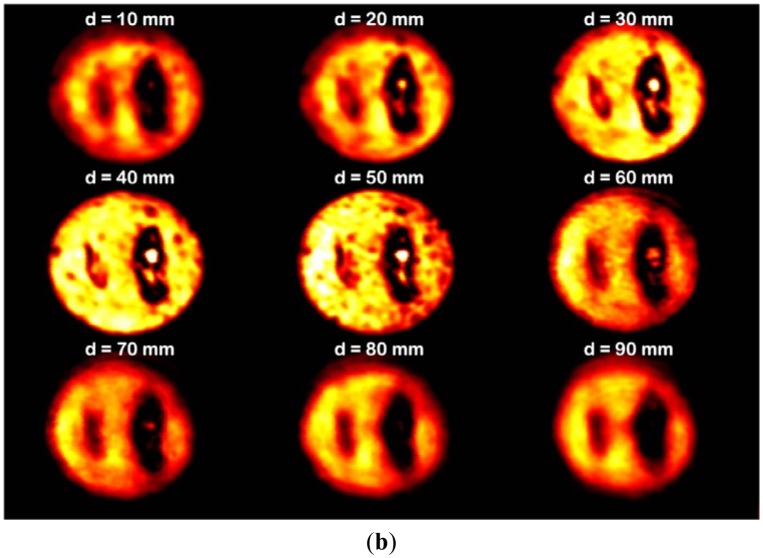
(**a**) CW terahertz transmission images for the Gaussian beam; (**b**) CW terahertz transmission images for the QBB. The color of each pixel in all images represents the intensity of transmitted beam, where the white color as a maximum intensity, the yellow color, the red color, and the black color as a minimum intensity were sequentially used for illustrating the intensity as arbitrary units. The diameter of circular image is the same as that of the sample (50 mm). In Figure 8, d is the distance from the flat lens surface or the apex of an axicon to sample surface.

To enable comparison with the image of a Gaussian beam using a conventional lens, a second TPX lens was placed in the same position as the axicon, as described in the previous subsection. Transmitted images of the sample using the Gaussian beam and the QBB are depicted in Figure 12(a,b), respectively. Each image was obtained using a sequence of sample positions from the second lens surface (or the axicon apex) in the propagation direction, the position varying from 10 mm to 90 mm in steps of 10 mm. For Gaussian beam illumination, a clear transmitted image was observed near the best focal length position (f = 38 mm), even though the images exhibited blurred edges and a distorted shape for the cricket at defocused positions.

The image formed by the QBB shows that the shape of objects is well preserved, in contrast to the image using the Gaussian beam. Importantly, a small object in the QBB image is distinguishable over the entire range of sample positions. These results are in good agreement with the characteristics of the 2D spatial-intensity distribution for the QBB in the propagation direction, as shown in Figure 2 and Figure 10(b). These results indicate that the QBB's property of extended depth of focus can be very useful for transmitted imaging over long working distances. It can be useful for quality inspection of homogeneous and smooth surfaced foods that requires noninvasive and safe terahertz technology.

## 5. Conclusions

To investigate the feasibility of using QBB imaging for noninvasive food inspection, we performed measurements on a food sample (noodle flour) containing embedded foreign objects, namely crickets. The transmitted QBB imaging shows that its property of extended depth of focus can enable long working distances for food-inspection applications and an efficient spatial resolution.

According to the numerical evaluation of beam profiles, the results of the FDTD method were different from those of analytical calculations based on scalar diffraction theory, in terahertz frequencies. To overcome these inaccuracies, new theoretical approaches should be studied for more precise description of beam-propagation properties in terahertz frequencies, including the wavefront effect, even though a rigorous FDTD calculation can give us better estimates and fruitful explanation.

Using the knife edge method, we obtained the minimum spot size of Gaussian beam and QBB. However, the beam profiles of QBB by the knife-edge method cannot describe the fundamental characteristics of QBB like the interference in central core. For obtaining more precise beam profiles over entire DOF range, the point scanning method was used for the characterization of QBB. Particularly, we found that the wavefront shape in the injection plane can strongly affect the performance of QBB, as a result of comparison between the beam profiles by point scanning and those of FDTD simulation. By developing high-speed and broad band detection over large sample areas, QBB imaging based on terahertz technology appears to be a promising technique for future noninvasive inspection applications. More detailed experimental and theoretical investigations should be performed in the near future for confirming the wavefront effect on the performance of QBB and overcoming the scattering effect caused by non-homogeneous media.
